# Consideration of the reference value and number of measurements of the urinary sodium-to-potassium ratio based on the prevalence of untreated home hypertension: TMM Cohort Study

**DOI:** 10.1038/s41440-021-00843-7

**Published:** 2022-01-18

**Authors:** Mana Kogure, Tomohiro Nakamura, Naho Tsuchiya, Takumi Hirata, Kotaro Nochioka, Akira Narita, Rieko Hatanaka, Fumi Itabashi, Ikumi Kanno, Taku Obara, Michihiro Satoh, Hirohito Metoki, Ken Miyagawa, Hiroshi Koshimizu, Sho Nagayoshi, Akira Uruno, Masahiro Kikuya, Kichiya Suzuki, Naoki Nakaya, Junichi Sugawara, Shinichi Kuriyama, Ichiro Tsuji, Shigeo Kure, Atsushi Hozawa

**Affiliations:** 1 Tohoku Medical Megabank Organization, Tohoku University, Sendai, Japan; 2 Graduate School of Medicine, Tohoku University, Sendai, Japan; 3 Yamato Home Medical Care Clinic Kurihara, Kurihara, Japan; 4 Hokkaido University Faculty of Medicine, Sapporo, Japan; 5 Tohoku University Hospital, Tohoku University, Sendai, Japan; 6 Tohoku Medical and Pharmaceutical University, Sendai, Japan; 7 OMRON Healthcare Co., Ltd, Kyoto, Japan; 8 Teikyo University School of Medicine, Tokyo, Japan; 9 International Research Institute of Disaster Science, Tohoku University, Sendai, Japan

**Keywords:** Home hypertension, Morning urinary Na/K ratio, Multiple measurements, Reference value, TMM Cohort Study

## Abstract

The sodium-to-potassium (Na/K) ratio is known to be associated with blood pressure (BP). However, no reference value has been established since the urinary Na/K (uNa/K) ratio is known to have diurnal and day-to-day variations. Therefore, we investigated the number of days required to yield a better association between the morning uNa/K ratio and home BP (HBP) and determined a morning uNa/K ratio value that can be used as a reference value in participants who are not taking antihypertensive medication. This was a cross-sectional study using data from the Tohoku Medical Megabank Project Cohort Study. A total of 3122 participants borrowed HBP and uNa/K ratio monitors for 10 consecutive days. We assessed the relationship between the morning uNa/K ratio from 1 day to 10 days and home hypertension (HBP ≥ 135/85 mmHg) using multiple logistic regression models. Although a 1-day measurement of the morning uNa/K ratio was positively associated with home hypertension, multiple measurements of the morning uNa/K ratio were strongly related to home hypertension. The average morning uNa/K ratio was relatively stable after 3 days (adjusted odds ratio of home hypertension per unit increase in the uNa/K ratio for more than 3 days: 1.19–1.23). In conclusion, there was no threshold for the uNa/K ratio, and the morning uNa/K ratio was linearly associated with home hypertension. The Na/K ratio 2.0 calculated from the Dietary Reference Intakes for Japanese might be a good indication. Regarding the stability of the association between the morning uNa/K ratio and BP, more than 3 days of measurements is desirable.

## Introduction

High blood pressure (BP) leads to major health risks, such as heart disease and stroke [1]. In Japan, the estimated number of people with hypertension in 2010 was 43 million [2]. Therefore, the prevention of hypertension is a crucial issue.

It is widely reported that high sodium (Na) and potassium (K) intake are known to be related to high and low BP, respectively [3–7]. In recent years, the balance between Na and K intake (Na/K (mol) ratio) has received significant attention. It has been reported that the Na/K ratio is associated with BP [8–13].

**Fig. 1:**
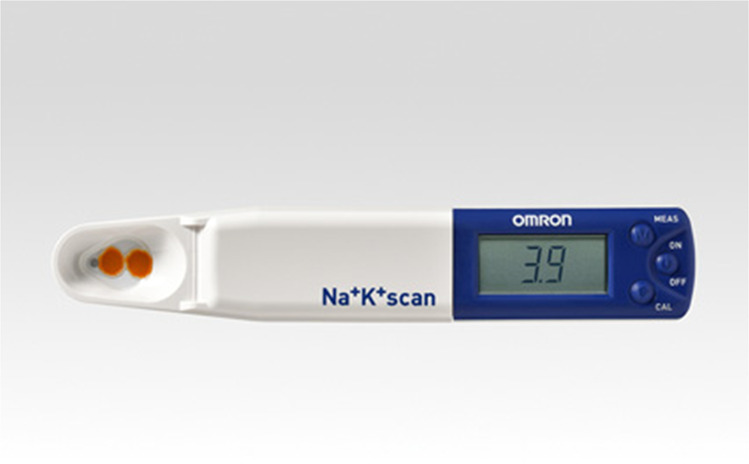
Handy-sized urinary Na/K ratio monitor (HEU-001F; OMRON Healthcare Co., Ltd., Kyoto, Japan)

Recently, OMRON Healthcare Co., Ltd. (Fig. 1, HEU-001F; OMRON Healthcare Co., Ltd., Kyoto, Japan) developed a handy-sized urinary Na/K ratio monitor that can quickly and easily measure the Na/K ratio in urine samples. If a handy-sized monitor can be used, it is expected that the urinary Na/K ratio can be controlled at home or during a health checkup. Therefore, it is necessary to consider a reference value of the urinary Na/K ratio.

However, the urinary Na/K ratio is known to have diurnal and day-to-day variations, and it is difficult to set the reference value for the casual urinary Na/K ratio. A previous study reported that the casual urinary Na/K ratio was higher in the morning and evening than during the daytime [14]. For day-to-day variation, another study showed that although a single measurement of the urinary Na/K ratio fairly correlated with seven consecutive 24-h urine samples, six random casual urine samples on different days were well correlated with Na/K ratios from seven consecutive 24-h urine samples [15]. This finding was also supported by our previous report that multiple measurements of the Na/K ratio correlated with home hypertension better than a single measurement [12].

Thus, to overcome the limitations of diurnal and day-to-day variations, the following two points should be considered: (1) information should be obtained on the urinary Na/K ratio with limited measurement times and (2) information should be obtained on the urinary Na/K ratio of multiple measurements. However, there have been no reports of large populations that have satisfied these points.

Thus, in this study, we investigated the number of days required to yield a better association between urinary Na/K ratio measurements in the morning and BP and what morning urinary Na/K ratio value can be used as a reference value in participants who are not taking antihypertensive medication.

## Methods

### Participant recruitment

This study was a cross-sectional study using data from the Tohoku Medical Megabank Project Cohort Study (TMM Cohort Study), which in 2011, aimed to comprehend and address the mental and physical impacts of the Great East Japan Earthquake. Furthermore, this study sought to improve health and medical care in this community. All participants were community residents of the Miyagi or Iwate prefectures and were recruited for this study between May 2013 and March 2016 (Approval number of the Ethical Review Board: 2019-4-065, HG H25-2) [16, 17].

Those who participated in the baseline survey of the TMM Cohort Study were invited to complete a secondary survey (i.e., Repeat assessment center-based survey during second period). Starting in June 2017, the participants could visit any of the seven Community Support Centers within Miyagi Prefecture to complete Repeat assessment center-based survey during second period. We also began a collaborative study in June 2017 with OMRON Healthcare Co., Ltd. for participants in Repeat assessment center-based survey during second period (June 2017–March 2021). At four of the seven Community Support Centers, we loaned urinary Na/K ratio monitors and BP measuring devices to the participants (three centers started in June 2017, and one center started in June 2020). Of the 15,554 participants who agreed to Repeat assessment center-based survey during second period at three Community Support Centers before 7 April 2020, 7887 participants agreed to participate in the collaborative study with OMRON Healthcare Co., Ltd. (response rate: 50.7%). All participants provided written, informed consent to participate in this study, as approved by the Institutional Review Board of Tohoku Medical Megabank Organization (Approval number: 2020-4-026).

### Inclusion/exclusion criteria

Of the 7887 participants who borrowed both urinary Na/K ratio monitors and BP measuring devices, those with no data on morning urinary Na/K ratios for 10 consecutive days (*n* = 3564), home blood pressure (HBP) in the morning (*n* = 22), information on treatment for hypertension (*n* = 146), listed height or weight (*n* = 14), and reported alcohol status (*n* = 16) were excluded from the analyses. Additionally, we excluded participants who were under treatment for hypertension (*n* = 1003). As a result, 3122 participants fulfilled all the criteria and had their data included in the analyses.

### Urinary Na/K ratio data collection

In this survey, participants were provided handy-sized urinary Na/K ratio monitors (HEU-001F; OMRON Healthcare Co., Ltd., Kyoto, Japan) for 10 days. This monitor can be used as a noninvasive, self-monitoring device for assessing the urinary Na/K ratio [18, 19]. These monitors automatically recorded the Na/K ratio measurement values in the device’s memory. The participants measured urinary Na/K ratios by themselves in the morning after waking up and in the evening before going to sleep. The staff at each Community Support Center provided face-to-face instructions on how to use the urinary Na/K ratio monitors to each participant; the participants were also requested not to lend the monitor to any other person. In addition, the staff calibrated and checked the sensitivity of the sensors before providing the Na/K ratio monitors to the participants. The authors conducted a preliminary validation test of the Na/K ratio monitor before the start of this study. The quality criteria of the Na/K ratio monitor is that the standard deviation (S.D.) of the calibration solution (Na/K = 4.0) measured after sensor calibration is within ±10%. In the preliminary validation test, six Na/K monitors were calibrated only on the first day, and the calibration solution (Na/K = 4.0) was measured for 10 days without calibration. As a consequence, the mean value of the Na/K ratio was 3.97 ± 0.29 (i.e., the S.D. was ±7.3%, unpublished data). From the above, it was confirmed that the result was within the range of quality assurance even if the calibration was conducted only on the first day. The monitors were returned to the Community Support Center by mail, and data from the monitors were uploaded to the Tohoku Medical Megabank Organization (ToMMo) network.

### HBP data collection

In this study, the participants were provided BP monitors (HEM-7080IC; OMRON Healthcare Co., Ltd., Kyoto, Japan) at home for 10 days during the same period of measuring urinary Na/K ratios. The participants personally measured their HBP in the morning after waking up and in the evening before going to sleep. In the morning, the participants were directed to take their HBP within 1 h after waking up, after urination, and before taking drugs or eating breakfast. In the evening, the participants measured their HBP in a seated position before going to sleep. After 10 days, the participants returned the BP monitors to the Community Support Center by mail. The BP monitor data were uploaded to the ToMMo network. The average HBP in the morning for 10 days was used for all analyses. Information on treatment for hypertension was obtained using a self-report questionnaire. The participants answered one question with the following options: 1. Ever diagnosed with hypertension [(a) under treatment for hypertension, (b) withdrawal of hypertension treatment, (c) under lifestyle modification without medication, and (d) under observation without medication] or 2. Has never been diagnosed with hypertension. In this study, the participants using antihypertensive medication were excluded from the analysis because their Na/K ratio values would be strongly affected by antihypertensive medication. Home hypertension was defined as a systolic BP (SBP) ≥ 135 mmHg and/or a diastolic BP (DBP) ≥ 85 mmHg [7].

### Covariate factors

We included potential confounding factors such as age, sex, body mass index (BMI), and drinking status. Age was determined at the time of consent for the secondary survey. Sex was identified using the information provided in the consent form. BMI was calculated from the height and weight obtained from the self-administered questionnaire. BMI was calculated using the following formula: weight (kg)/height (m^2^).

Alcohol drinking status (frequency and amount per day) was determined using a self-administered questionnaire and was classified into the following four categories: current-drinker, ex-drinker, never-drinker, and unable to drink constitutionally. The type of alcohol was classified into the following six categories: sake, distilled spirits, shochu-based beverages, beer, whiskey, and wine. The frequency of alcohol intake was classified into the following six categories: almost never, 1–3 days/month, 1–2 days/week, 3–4 days/week, 5–6 days/week, or daily. The participants answered how much of each type of alcohol they drank. Each type of alcohol intake was multiplied by the frequency and amount and converted to ethanol. The drinking amount was classified into the following four categories: never-drinker, ex-drinker, <23 g per day, and ≥23 g per day. We determined the cutoff value of the alcohol amount to be 23 g because it is the traditional Japanese unit of sake.

### Calculations and statistical analysis

In this study, we used morning urinary Na/K ratio data. It has been reported that urination is more frequent in the morning than at other times of the day under free-living conditions in the wakeful state [14]. Moreover, it was also reported that the most common time of day for home BP monitoring in patients under treatment for hypertension was in the morning [20]. Moreover, most patients with lifestyle-related diseases in Japan seem to take their daily medication in the morning hours despite there being no restrictions on taking medication at a specific time slot [21].

As mentioned above, if the urinary Na/K ratio monitor is introduced in households as a self-monitoring tool in the future, it is expected that some measurements will be taken in the morning in conjunction with a home BP monitor. We used the average morning urinary Na/K ratio from 1 day to 10 days. The daily morning urinary Na/K ratio was calculated as the average morning urinary Na/K ratios. If multiple measurements of the urinary Na/K ratio were taken at the same time of day, the first measurement value was adopted. Measurements from 4 a.m. to 10 a.m. were taken as morning measurements.

Regarding the baseline characteristics, we classified participants’ average morning urinary Na/K ratios at 10 days into nine categories (<1.0, 1.0–1.9, 2.0–2.9, 3.0–3.9, 4.0–4.9, 5.0–5.9, 6.0–6.9, 7.0–7.9, and ≥8.0). We tested for trends in the morning urinary Na/K ratio based on participants’ characteristics, including age, sex, BMI, morning urinary Na/K ratios for 10 days, SBP, DBP, drinking status, home hypertension, and information on treatment for hypertension. We performed trend tests to evaluate the linear relationship between the morning urinary Na/K ratio and the variables mentioned above. We used a general linear model for the continuous variables of age, BMI, morning urinary Na/K ratio for 10 days, SBP, and DBP and a logistic regression model for the categorical variables of sex, drinking status, home hypertension, and information on treatment for hypertension.

To examine the relationship between morning urinary Na/K ratio for 10 days and HBP in detail, we performed an analysis of covariance to calculate the adjusted mean HBP. The morning urinary Na/K ratio was classified into nine categories. Additionally, we stratified the participants into two groups: ever diagnosed with hypertension (*n* = 496) and never diagnosed with hypertension (*n* = 2628) to consider the influence of treatment status on hypertension. Furthermore, we tested for linear trends in HBP according to the nine categories of morning urinary Na/K ratios. In this analysis, we included age, BMI, and alcohol intake as potential confounding factors.

To examine the relationship between the morning urinary Na/K ratio from 1 day to 10 days and home hypertension, we used multiple logistic regression models and calculated adjusted odds ratios (aORs) with 95% confidence intervals. The morning urinary Na/K ratio was classified into eight categories (<2.0, 2.0–2.9, 3.0–3.9, 4.0–4.9, 5.0–5.9, 6.0–6.9, 7.0–7.9, and ≥8.0) because there were a few participants with a morning urinary Na/K ratio of less than 1.0. Adjusted odds ratio *P* values for linear trends were calculated using the nine categories of urinary Na/K ratios. We included potential confounding factors in the model, such as age, sex, BMI, and drinking status.

We calculated the area under the receiver operating characteristic curve (AUROC) to assess whether multiple measurements of morning urinary Na/K ratios increased the ability to predict home hypertension.

Furthermore, to consider the reference value of the urinary Na/K ratio, the salt equivalent and potassium intake, which are the tentative dietary goal for preventing life-style related diseases (DG) in the Dietary Reference Intakes for Japanese, 2020 [22], were converted into mEq/day, and the urinary Na/K ratio was calculated. The urinary excretion of Na and K was calculated assuming 86% and 77% of the total intake, respectively [23].

All analyses were performed using SAS version 9.4 for Windows (SAS Inc., Cary, NC, USA).

## Results

We analyzed 820 men and 2302 women who did not take antihypertensive medication. Table 1 shows the baseline characteristics of the participants not taking antihypertensive medication grouped by their average 10-day morning urinary Na/K ratio. Younger participants, men, those with a higher BMI, those with a higher SBP, those with a higher DBP, and current drinkers (≥23 g/day) were more likely to have an increased morning urinary Na/K ratio (*P* for linear trend <0.05). The proportion of participants who had ever been diagnosed with hypertension was 50.0% in the group with a morning urinary Na/K ratio <1.0.

**Table 1:** Characteristics of study participants with untreated hypertension according to morning urinary Na/K ratio

	Morning urinary Na/K ratio for 10 days									
	<1.0	1.0–1.9	2.0–2.9	3.0–3.9	4.0–4.9	5.0–5.9	6.0–6.9	7.0–7.9	≥8.0	*P* for linear trend
Numbers	6	57	415	787	752	534	266	158	147	
Age (years) (means ± SD)	54.2 ± 13.2	59.0 ± 11.1	59.1 ± 13.1	58.5 ± 13.5	57.4 ± 13.5	56.8 ± 13.9	57.3 ± 14.1	56.4 ± 12.8	58.7 ± 13.5	0.033
Sex (number, %)										
Men	0 (0.0)	10 (17.5)	95 (22.9)	178 (22.6)	179 (23.8)	147 (27.5)	88 (33.1)	59 (37.3)	64 (43.5)	<0.001
Women	6 (100.0)	47 (82.5)	320 (77.1)	609 (77.4)	573 (76.2)	387 (72.5)	178 (66.9)	99 (62.7)	83 (56.5)	<0.001
BMI (kg/m^2^) (means ± SD)	21.1 ± 1.8	22.4 ± 3.8	22.2 ± 2.9	22.6 ± 3.2	22.6 ± 3.2	22.8 ± 3.2	22.9 ± 3.3	23.5 ± 3.6	22.8 ± 3.1	<0.001
Morning urinary Na/K ratio for 10 days (means ± SD)	0.8 ± 0.2	1.7 ± 0.2	2.6 ± 0.3	3.5 ± 0.3	4.5 ± 0.3	5.5 ± 0.3	6.4 ± 0.3	7.4 ± 0.3	9.8 ± 2.1	<0.001
SBP (mmHg) (means ± SD)	122.9 ± 25.8	117.5 ± 11.9	119.2 ± 13.8	121.7 ± 15.3	121.3 ± 15.1	123.8 ± 16.3	125.9 ± 17.8	128.6 ± 17.5	130.5 ± 18.8	<0.001
DBP (mmHg) (means ± SD)	73.5 ± 13.4	71.4 ± 8.8	72.3 ± 8.9	73.0 ± 9.7	73.2 ± 9.6	74.6 ± 9.3	76.0 ± 10.2	77.6 ± 10.9	78.9 ± 11.4	<0.001
Drinking status (number, %)										
Never-drinker	4 (66.7)	28 (49.1)	232 (55.9)	400 (50.8)	378 (50.3)	290 (54.3)	127 (47.7)	75 (47.5)	68 (46.3)	0.063
Ex-drinker	0 (0.0)	3 (5.3)	13 (3.1)	18 (2.3)	23 (3.1)	8 (1.5)	8 (3.0)	3 (1.9)	2 (1.4)	0.165
<23 g/day	1 (16.7)	21 (36.8)	119 (28.7)	267 (33.9)	254 (33.8)	157 (29.4)	81 (30.5)	46 (29.1)	45 (30.6)	0.408
≥23 g/day	1 (16.7)	5 (8.8)	51 (12.3)	102 (13.0)	97 (12.9)	79 (14.8)	50 (18.8)	34 (21.5)	32 (21.8)	<0.001
Home hypretension^a^ (number, %)	2 (33.3)	7 (12.3)	62 (14.9)	181 (23.0)	149 (19.8)	124 (23.2)	93 (35.0)	59 (37.3)	62 (42.2)	<0.001
Information on treatment for hypertension (number, %)										
Discontinued treatment										
Yes	1 (16.7)	3 (5.3)	9 (2.2)	18 (2.3)	12 (1.6)	9 (1.7)	2 (0.8)	2 (1.3)	4 (2.7)	0.111
Undertaking lifestyle modification without medication										
Yes	1 (16.7)	7 (12.3)	27 (6.5)	55 (7.0)	53 (7.1)	31 (5.8)	17 (6.4)	13 (8.2)	19 (12.9)	0.321
Under observation without medication										
Yes	1 (16.7)	3 (5.3)	18 (4.3)	36 (4.6)	30 (4.0)	31 (5.8)	15 (5.6)	11 (7.0)	9 (6.1)	0.139
Ever been diagnosed with hypertension										
Yes	3 (50.0)	16 (28.1)	62 (14.9)	129 (16.4)	106 (14.1)	80 (15.0)	36 (13.5)	30 (19.0)	34 (23.1)	0.659

*BMI* body mass index, *DBP* diastolic blood pressure, *Na/K ratio* sodium/potassium ratio, *SBP* systolic blood pressure, *SD* standard deviation

^a^Home hypertension was defined as an SBP ≥ 135 mmHg and/or a DBP ≥ 85 mmHg.

**Fig. 2:**
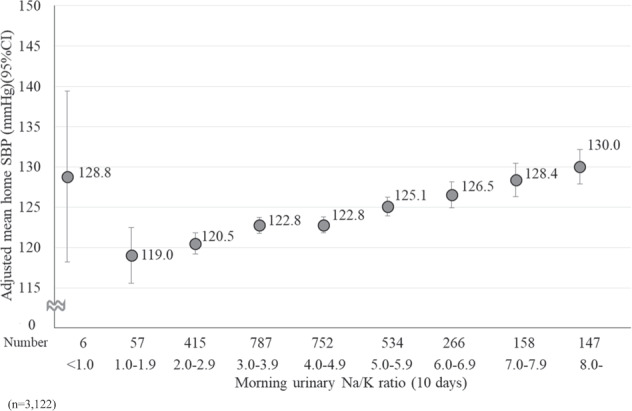
The relationship between the 10-day average morning urinary Na/K ratio and the adjusted mean home SBP. The adjusted mean home SBP was calculated from the analysis of covariance. The error bars represent a 95% confidence interval

Figure 2 shows the relationship between the 10-day average morning urinary Na/K ratio and the adjusted mean home SBP. Although home SBP was high in the group with a morning urinary Na/K ratio <1.0, it was positively associated with the morning urinary Na/K ratio (*P* for linear trend <0.001).

**Fig. 3:**
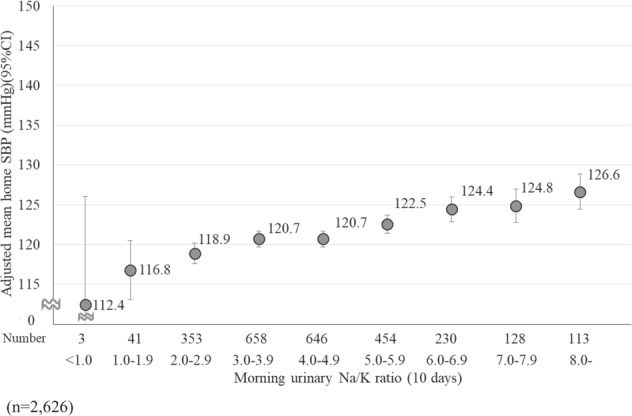
The relationship between the 10-day average morning urinary Na/K ratio and the adjusted mean home SBP among the 2626 participants who had never been diagnosed with hypertension. The adjusted mean home SBP was calculated from the analysis of covariance. The error bars represent a 95% confidence interval

Figure 3 shows the relationship between the 10-day average morning urinary Na/K ratio and the adjusted mean home SBP among the 2626 participants who never been diagnosed with hypertension. The morning urinary Na/K ratio was positively associated with home SBP (*P* for linear trend <0.001).

**Fig. 4:**
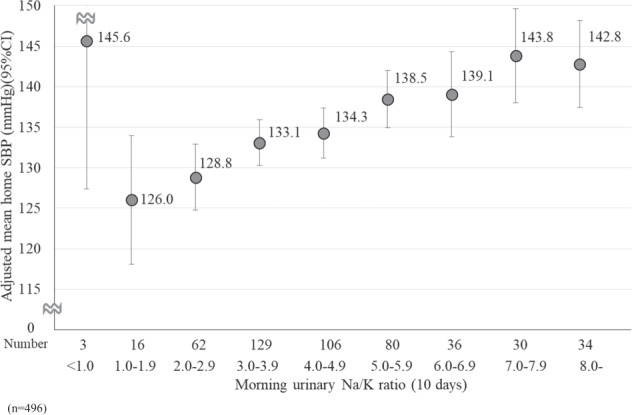
The relationship between the 10-day average morning urinary Na/K ratio and the adjusted mean home SBP among the 496 participants who had ever been diagnosed with hypertension. The adjusted mean home SBP was calculated from the analysis of covariance. The error bars represent a 95% confidence interval

Figure 4 shows the relationship between the 10-day average morning urinary Na/K ratio and the adjusted mean home SBP among the 496 participants who had ever been diagnosed with hypertension. Although home SBP was high in the group with a morning urinary Na/K ratio <1.0, it was positively associated with the morning urinary Na/K ratio (*P* for linear trend <0.001).

Table 2 shows the relationship between the average morning urinary Na/K ratios from Day 1 to Day 10 and home hypertension. Although a 1-day measurement of the morning urinary Na/K ratio was positively associated with home hypertension, multiple measurements of the morning urinary Na/K ratio were related to home hypertension. In addition, the aOR of home hypertension per unit increase in the average morning urinary Na/K ratio increased from Day 1 to Day 3; however, the aOR was stable after 3 days (range 1.19–1.23). Similarly, the AUROC was increased according to the measurement days. The AUROC was almost stable after 3 days (AUROC = 0.764–0.768). The AUROC without the morning urinary Na/K ratio was 0.753.

**Table 2:** Relationship between 1 day and 10 days average morning urinary Na/K ratios and home hypertension among 3,122 participants without taking antihypertensive medication

Multiple adjusted OR (95% CI)											
Morning urinary Na/K ratio											
	<2.0	2.0–2.9	3.0–3.9	4.0–4.9	5.0–5.9	6.0–6.9	7.0–7.9	≥8.0	*P* for linear trend	OR (95% CI)^a^	Area under the ROC curve^b^
1 day											
Number of home hypertension^c^/number of participants	42/228	106/556	170/678	131/604	99/426	70/271	40/144	81/215			
	Ref.	0.89 (0.58–1.36)	1.38 (0.92–2.07)	1.11 (0.73–1.69)	1.23 (0.80–1.90)	1.50 (0.94–2.40)	1.60 (0.94–2.75)	2.55 (1.58–4.11)	< 0.001	1.12 (1.07–1.18)	0.761
2 days											
Number of home hypertension/number of participants	21/138	100/499	151/731	152/664	119/479	74/254	54/165	68/192			
	Ref.	1.21 (0.70–2.09)	1.42 (0.84–2.41)	1.43 (0.84–2.43)	1.69 (0.98–2.90)	2.10 (1.18–3.74)	2.70 (1.47–4.97)	2.80 (1.54–5.07)	<0.001	1.15 (1.10–1.21)	0.760
3 days											
Number of home hypertension/number of participants	15/108	86/465	160/758	150/696	123/476	78/288	56/144	71/187			
	Ref.	1.34 (0.71–2.51)	1.66 (0.90–3.06)	1.53 (0.83–2.82)	2.07 (1.11–3.86)	2.45 (1.28–4.69)	3.73 (1.86–7.48)	3.72 (1.91–7.26)	<0.001	1.19 (1.13–1.25)	0.765
4 days											
Number of home hypertension/number of participants	12/87	76/439	172/781	150/708	129/516	74/273	60/147	66/171			
	Ref.	1.25 (0.62–2.51)	1.82 (0.93–3.58)	1.57 (0.80–3.09)	2.05 (1.03–4.06)	2.20 (1.08–4.48)	4.44 (2.10–9.37)	3.74 (1.80–7.79)	<0.001	1.19 (1.13–1.25)	0.766
5 days											
Number of home hypertension/number of participants	15/79	68/443	169/781	157/714	128/512	75/284	61/150	66/159			
	Ref.	0.77 (0.40–1.50)	1.20 (0.64–2.26)	1.18 (0.63–2.22)	1.44 (0.76–2.74)	1.44 (0.73–2.82)	3.11 (1.54–6.30)	2.85 (1.41–5.74)	<0.001	1.20 (1.14–1.27)	0.766
6 days											
Number of home hypertension/number of participants	14/76	61/413	174/799	156/725	136/531	82/282	55/149	61/147			
	Ref.	0.71 (0.36–1.41)	1.17 (0.61–2.23)	1.09 (0.57–2.08)	1.45 (0.75–2.79)	1.72 (0.87–3.40)	2.31 (1.13–4.75)	2.77 (1.35–5.70)	<0.001	1.21 (1.14–1.27)	0.765
7 days											
Number of home hypertension/number of participants	12/76	64/413	169/779	167/756	129/530	85/284	49/131	64/153			
	Ref.	1.01 (0.49–2.07)	1.57 (0.79–3.11)	1.53 (0.77–3.03)	1.89 (0.95–3.79)	2.36 (1.15–4.83)	3.01 (1.40–6.50)	3.87 (1.83–8.18)	<0.001	1.21 (1.15–1.28)	0.764
8 days											
Number of home hypertension/number of participants	11/70	58/415	183/779	158/770	121/509	93/298	49/126	66/155			
	Ref.	0.79 (0.37–1.68)	1.55 (0.76–3.16)	1.27 (0.62–2.59)	1.68 (0.81–3.46)	2.33 (1.11–4.90)	2.66 (1.20–5.90)	3.47 (1.60–7.53)	<0.001	1.22 (1.15–1.28)	0.767
9 days											
Number of home hypertension/number of participants	12/67	57/415	179/782	161/767	118/525	96/277	51/139	65/150			
	Ref.	0.68 (0.32–1.41)	1.30 (0.65–2.62)	1.17 (0.58–2.37)	1.33 (0.65–2.70)	2.25 (1.09–4.67)	2.24 (1.03–4.88)	3.26 (1.52–7.00)	<0.001	1.23 (1.16–1.29)	0.768
10 days											
Number of home hypertension/number of participants	9/63	62/415	181/787	149/752	124/534	93/266	59/158	62/147			
	Ref.	0.95 (0.42–2.13)	1.64 (0.75–3.57)	1.39 (0.64–3.03)	1.69 (0.77–3.71)	2.98 (1.33–6.68)	3.06 (1.32–7.09)	3.84 (1.66–8.89)	<0.001	1.23 (1.16–1.29)	0.768

*BMI* body mass index, *BP* blood pressure, *Na/K ratio* sodium/potassium ratio, *OR* odds ratio, *Ref* reference, *95% CI* 95% confidence interval, *ROC* receiver operating characteristic curve

^a^The prevalence of home hypertension per unit increase in average urinary Na/K ratios from 1 to 10 days.

^b^Adjusted for age (continuous variable), sex, BMI (continuous variable), and drinking status (never drinker, ex–drinker, <23 g per day, and ≥23 g per day).

^c^Home hypertension was defined as an SBP ≥ 135 mmHg and/or a DBP ≥ 85 mmHg.

The urinary Na/K ratio was calculated as mEq/day based on Na intake and K intake, which are the DG in the Dietary Reference Intakes for Japanese, 2020. The DG of a salt equivalent was 7.5 g/day in adult men and 6.5 g/day in adult women. These values corresponded to 128.4 mEq/day and 111.3 mEq/day of Na, respectively. Similarly, the DG of K intake was 3000 mg/day in adult men and 2600 mg/day in women. These values corresponded to 76.7 mEq/day and 66.5 mEq/day, respectively. The urinary excretion of Na and K was calculated assuming 86% and 77% of the total intake, respectively (Na: 110.4 mEq/day, K: 59.1 mEq/day in men; Na: 95.7 mEq/day, K: 51.2 mEq/day in women). Thus, the urinary Na/K ratio was 1.87 for both men and women.

## Discussion

We investigated the number of days required to yield a better association between the urinary Na/K ratio measured in the morning and BP and what morning urinary Na/K ratio value can be used as a reference value in participants who are not taking antihypertensive medication.

Although a 1-day measurement of the morning urinary Na/K ratio was positively associated with home hypertension, multiple measurements of the morning urinary Na/K ratio were strongly related to home hypertension. In addition, although the relationship was stronger for measurements including more days, the aOR of home hypertension per unit increase in the average morning urinary Na/K ratio was relatively stable after 3 days. A previous study reported that the mean urinary Na/K ratio of 4–7 days on different days correlated with a 1–2 day 24-h urinary Na/K ratio with high BP [24]. Another study reported that the correlation coefficient increased up to 4 days, and the urinary Na/K ratio was relatively stable at ≥0.80 [15]. Our results were consistent with those of previous studies. There was no threshold for the urinary Na/K ratio, and the morning urinary Na/K ratio was linearly associated with home hypertension. However, if the urinary Na/K ratio measurement is introduced into home or health checkups in the future, a reference value for the urinary Na/K ratio is necessary.

The World Health Organization guidelines recommend an Na/K ratio of approximately less than 1.0 for health benefits [3, 4]. However, considering the salt and K intake in Japan, it is expected to be difficult to reduce the Na/K ratio to less than 1.0 in real life.

In contrast, the risk of cardiovascular disease has been reported to be low even at urinary Na/K ratio below 2.0 [25]. Furthermore, the urinary Na/K ratio, which is calculated by converting the urinary excretion of Na and K from the Na and K targeted intake in the Dietary Reference Intakes for Japanese, 2020, was approximately 2.0 for both men and women. Therefore, we consider it suitable to aim for a urinary Na/K ratio of less than 2.0 at this point.

It is necessary to consider how to define the reference value by using data on Na and K intake, which are set as target values in other guidelines, or by using the median value as the cutoff point based on the distribution of the urinary Na/K ratio measured multiple times in a large population, such as in this study.

Our results regarding the relationship between the urinary Na/K ratio and BP were consistent with those of previous studies [8–13]. However, although the difference was not statistically significant, home SBP was higher in the group with a morning urinary Na/K ratio less than 1.0 compared to the other groups’ morning urinary Na/K ratio. We considered reasons why home SBP was higher in the group with a morning urinary Na/K ratio less than 1.0. First, some medications might affect the urinary Na/K ratio and BP. The TMM Cohort Study obtained information about medication status (including the duration, frequency, and amount of medication used) using a self-report questionnaire. In terms of medication status, one participant in the group with a morning urinary Na/K ratio less than 1.0 who had ever been diagnosed with hypertension was taking glycyrrhizin supplements. It is also known that glycyrrhizin supplements may cause pseudoaldosteronism. These results suggested that home SBP was higher in the group with a morning urinary Na/K ratio less than 1.0. Previous reports have shown a high correlation between the urinary Na/K ratio and 24-h urinary Na/K ratio in chronic kidney disease (CKD) stages 1–3 but not in CKD stages 4–5 because some CKD stages 4–5 patients take diuretics that affect the urinary Na/K ratio; it is difficult to evaluate multiple measurements of the urinary Na/K ratio [26]. Furthermore, a recent study reported that lower urinary Na/K ratio might detect primary hyperaldosteronism [27]. Thus, we considered that participants with extremely low urinary Na/K ratio need to be cautious because they may be affected by medications. Another possible reason might be reverse causality; some participants with high BP might have already reduced their salt intake and/or increased their vegetable intake. This possibility was supported by Fig. 3, i.e., we excluded participants who had been diagnosed with hypertension in the past and showed a linear association between the morning urinary Na/K ratio and home SBP.

The present study has some strengths. First, this is the first study to investigate the reference value of the morning urinary Na/K ratio for hypertension in a large population using data on the Na/K ratio with a limited measurement time and on multiple measurements to consider diurnal variation and day-to-day variation. Finally, we included various confounding factors.

Our study also has some limitations. First, since this study had a cross-sectional design, it is difficult to confirm reverse causality. Participants with high BP might have developed good behaviors, such as reducing their salt and alcohol intake. Therefore, we considered that lifestyle modifications for high BP would lead to an underestimated relationship between a high Na/K ratio and home hypertension. Second, we could not consider seasonal variations in home BP and the urinary Na/K ratio in this study. It has been reported that the urinary Na/K ratio and home BP exhibit seasonal variations [28, 29]. If seasonal variations were considered in this analysis, the relationship between the urinary Na/K ratio and home BP might have been stronger. Finally, we did not directly assess whether the predictive power of the long-term casual urinary Na/K ratio might be equivalent to that of a 24-h urinary Na/K ratio as the gold standard. However, a single measurement of the urinary Na/K ratio fairly correlated with seven consecutive 24-h urine samples, and six random casual urine samples on different days were well correlated with Na/K ratios from seven consecutive 24-h urine samples [15]. Therefore, we believe that our findings are applicable.

In conclusion, we found that there was no threshold for the urinary Na/K ratio, and the morning urinary Na/K ratio was linearly associated with home hypertension. However, participants with extremely low urinary Na/K ratio need to be cautious in following the situation of the population. In addition, it is necessary to consider the reference value while defining a cutoff based on the distribution of the population and utilizing other guidelines. At present, the Na/K ratio of 2.0, calculated from the Dietary Reference Intakes for Japanese, 2020, might be a good indication. Regarding the stability of the association between the morning urinary Na/K ratio and BP, it would be desirable to measure it for more than 3 days.
